# Budgetary Impact of the Medicare Shared Savings Program on Traditional Medicare

**DOI:** 10.1001/jamahealthforum.2025.6915

**Published:** 2026-02-20

**Authors:** Dhruv Khullar, William L. Schpero, Yasin Civelek, Lawrence P. Casalino, Manyao Zhang, Reekarl Pierre, Amelia M. Bond

**Affiliations:** 1 Division of Health Policy and Economics, Weill Cornell Medical College, New York, New York; 2 Cornell Health Policy Center, New York, New York; 3 The Physicians Foundation Center for the Study of Physician Practice and Leadership, Weill Cornell Medicine, New York, New York; 4 Division of General Internal Medicine, Weill Cornell Medical College, New York, New York

## Abstract

This cross-sectional study provides updated estimates of the Medicare Shared Savings Program’s budgetary impact between 2012 and 2023 using a more recent longitudinal evaluation of the program’s effects.

## Introduction

The Medicare Shared Savings Program (MSSP), which began in 2012, makes incentive payments to groups of clinicians and health care organizations known as accountable care organizations (ACOs) that achieve spending reductions for attributed patients while meeting quality targets. A study by Ryan and Markovitz^1^ found that the program was associated with net losses (after incentive payments) to traditional Medicare of $584 million to $1.42 billion between 2013 and 2021, using estimates of ACO savings in the initial years of MSSP.^2,3^ In this cross-sectional study, we provide updated estimates of MSSP’s budgetary impact between 2012 and 2023 using a more recent longitudinal evaluation of the program’s effects.^4^

## Methods

To examine net MSSP-related changes in traditional Medicare spending between 2012 and 2023, we used publicly available data on MSSP beneficiary counts, spending, and Medicare payments (shared savings and losses), as well as estimates of differential spending changes (in percentage terms) associated with ACO formation. We applied the primary and most conservative savings estimates from a 2025 study by Bond et al,^4^ which examined ACO performance from 2012 to 2019 and found that ACOs achieved increasing spending reductions over time (ranging from 1.2%-1.9% annually in year 3 and 3.5%-4.8% annually in year 6) (eMethods in Supplement 1).

We calculated an ACO’s annual savings using percentage savings estimates for each year relative to ACO formation and the ACO’s overall spending in that year. We also extracted shared savings payments made by Medicare (and shared loss payments from ACOs to Medicare) in each year. Net savings represented the difference between gross spending reductions generated by ACOs and payments made or received by Medicare during the study period.

Spending was inflation adjusted to 2023 US dollars using the US Consumer Price Index. The institutional review board at Weill Cornell Medical College deemed this study exempt from review because it used publicly available and secondary, deidentified data. This study adheres to the Strengthening the Reporting of Observational Studies in Epidemiology (STROBE) reporting guidelines.

## Results

In models using both primary and conservative savings estimates, ACOs generated net savings to Medicare beginning in the second year of the program (Table). Annual per-beneficiary net savings increased from $20 to $33 in 2014 to $107 to $208 in 2018. Net savings fluctuated between 2019 and 2023, ranging from $0 in 2020 (conservative model) to $202 in 2021 (primary model). Between 2012 and 2023, MSSP ACOs produced an estimated $20.1 billion to $29.2 billion in gross savings, and Medicare made $15.8 billion in bonus payments, resulting in net savings to traditional Medicare of $4.3 billion to $13.4 billion (Figure).

**Table.** Medicare Shared Savings Program (MSSP) Payments, Savings, Tenure, and Beneficiary Count by Year

Year	Medicare payments to ACOs, $ per beneficiary^a^	ACO savings relative to non-ACOs, $ per beneficiary^b^		Net Medicare savings, $ per beneficiary^c^		ACO tenure, mean (SE), y	Total MSSP beneficiaries
		Primary estimate	Conservative estimate	Primary estimate	Conservative estimate		
2013	111	79	90	−32	−21	1 (0)	3 675 263
2014	81	114	101	33	20	1.7 (0.5)	5 329 831
2015	109	176	122	67	13	2.3 (0.8)	7 270 233
2016	104	231	147	127	43	2.9 (1.1)	7 884 058
2017	100	266	169	166	69	3.3 (1.5)	8 992 886
2018	108	316	215	208	107	3.6 (1.8)	10 096 874
2019	123	296	201	173	78	4.4 (1.9)	9 997 705
2020	228	333	228	105	0	4.5 (2.4)	10 614 589
2021	202	404	274	202	72	5.4 (2.4)	10 124 325
2022	243	411	284	168	41	5.8 (3.0)	10 418 297
2023	301	456	318	155	17	6.3 (3.2)	10 235 411

Abbreviation: ACO, accountable care organization.

^a^ Medicare payment represents the shared savings payments made by Medicare (and shared loss payments from ACOs to Medicare). Payments were inflation adjusted to 2023 US dollars using the US Consumer Price Index.

^b^ ACO savings are average annual estimates from Bond et al^4^ weighted by MSSP beneficiaries for a given ACO tenure. Note that ACO savings increase as average ACO tenure increases. Estimates were inflation adjusted to 2023 US dollars using the US Consumer Price Index.

^c^ Net Medicare savings is the difference between ACO savings and Medicare payments to ACOs.

**Figure.**
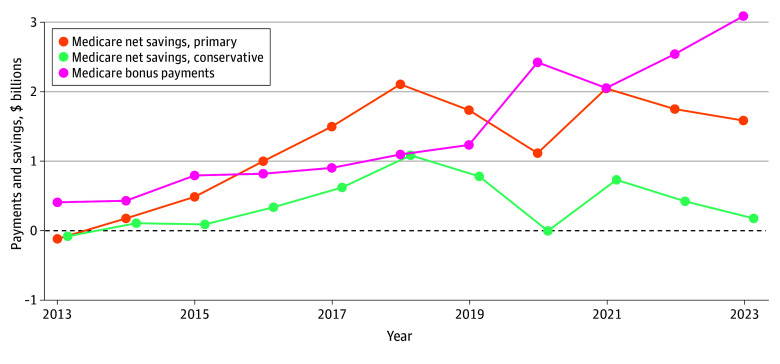
Medicare Shared Savings Program Payments and Net Medicare Savings by Year, 2013-2023 This line graph plots total Medicare payments to accountable care organizations (pink line), as well as net Medicare savings across all accountable care organizations by year. Net Medicare savings is calculated using primary estimates (orange) and more conservative estimates (green).

## Discussion

In this cross-sectional study using recent longitudinal spending estimates, we found that MSSP was associated with net savings of $4.3 billion to $13.4 billion to traditional Medicare between 2012 and 2023. These findings suggest that MSSP had a positive budgetary impact during the program’s first decade, contrasting with prior work that used older estimates and found net losses to Medicare.^1^ This difference likely results from the fact that Bond et al^4^ examined ACO performance over a longer time period compared to previous studies and found that savings grew as ACO tenure increased. Although MSSP generated net savings in most years, the introduction of regional benchmarks and reduced health care spending during the COVID-19 pandemic likely resulted in the relatively lower savings after 2019.

Limitations of this study include the inability to account for costs related to administering MSSP and the projection of earlier estimates through 2023. Nonetheless, these findings may be of interest to policymakers considering the budgetary effects of value-based payment reforms.

## Notes

Supplement 1.
eMethods

Supplement 2.
Data Sharing Statement

## References

[1] Ryan AM, Markovitz AA. Estimated savings from the Medicare Shared Savings Program. JAMA Health Forum. 2023;4(12):e234449. Retracted and replaced in: JAMA Health Forum. 2024;5(4):e240043. doi:10.1001/jamahealthforum.2023.4449 38100095 PMC10724775

[2] McWilliams JM, Hatfield LA, Landon BE, Hamed P, Chernew ME. Medicare spending after 3 years of the Medicare Shared Savings Program. N Engl J Med. 2018;379(12):1139-1149. doi:10.1056/NEJMsa1803388 30183495 PMC6269647

[3] Assessing the Medicare Shared Savings Program’s effect on Medicare spending. Medicare Payment Advisory Commission. June 2019. Accessed January 20, 2026. https://www.medpac.gov/wp-content/uploads/import_data/scrape_files/docs/default-source/reports/jun19_ch6_medpac_reporttocongress_sec.pdf

[4] Bond AM, Civelek Y, Schpero WL, . Long-term spending of accountable care organizations in the Medicare Shared Savings Program. JAMA. 2025;333(21):1897-1905. doi:10.1001/jama.2025.3870 40293760 PMC12038717

