# Supplementary material for: Budgetary Impact of the Medicare Shared Savings Program on Traditional Medicare
Source: JAMA Health Forum. 2026 Feb 20;7(2):e256915. doi: 10.1001/jamahealthforum.2025.6915 (PMC12924104; doi:10.1001/jamahealthforum.2025.6915)
Supplement: Supplement 1. — eMethods [file jamahealthforum-e256915-s001.pdf]

Supplemental Online Content

Khullar D, Schpero WL, Civelek Y, et al. Budgetary impact of the Medicare Shared Savings Program on traditional Medicare. *JAMA Health Forum*. 2026;7(2):e256915. doi:10.1001/jamahealthforum.2025.6915

eMethods

This supplemental material has been provided by the authors to give readers additional information about their work.

© 2026 Khullar D et al. *JAMA Health Forum*.

eMethods

Estimating net savings

We adapted the methodology developed by Ryan and Markovitz (2022) to estimate net savings using publicly reported data on MSSP from 2012-2023.[1] Specifically, we applied the estimates from Bond et al (2025) as percent total Medicare savings by year to publicly reported data on MSSP participant tenure, total spending, and Medicare Payment to ACOs to estimate MSSP savings.[2] Below is the specific equation we applied:

$$Total\ Savings = \sum_{ACO} \sum_{K} \alpha_k\ x\ Total\ ACO\ Spending_{ak} - Medicare\ Payment\ to\ ACO_{ak}$$

Where *a* is an MSSP ACO, *k* is the year relative to ACO formation, and $\alpha_k$comes from the equation above and represents the percent cost or savings associated with ACO formation in the kth year of MSSP ACO participation. For cohort that entered the MSSP in 2012, 2019, and 2021, we conservatively considered the 1st year of full ACO participation to be in 2013, 2020, and 2022 respectively, since the ACO cohorts began participation in March or July of the first year. Annual savings estimates and Medicare payment (shared savings or losses) were inflated to 2023 dollars using Medical CPI.

This paper used two sets of α_k estimates from Bond et al. The **main estimates** controls for patient covariates as well as ACO fixed effects and HRR by year fixed effects. The **conservative estimates** mitigate the extent to which patient selection may drive results by replacing all patient-level covariates with patient fixed effects.

Note that average spending among ACO cohorts declined in the later years of MSSP, which observers have attributed to the regionalization of benchmarks. Organizations with lower spending compared to others in their region may have faced relative high spending benchmarks, which may have incentivized low-spending ACOs to join the program. In order to account for the decline in average ACO spending over time, we applied savings estimates from Bond et al in percentages (rather than levels).

---

[1] Ryan AM. Notice of Retraction and Replacement. Ryan and Markovitz. Estimated Savings From the Medicare Shared Savings Program. JAMA Health Forum. 2023;4(12):e234449. JAMA Health Forum. 2024;5(4):e240043. doi:10.1001/jamahealthforum.2024.0043.

[2] Bond AM, Civelek Y, Schpero WL, et al. Long-Term Spending of Accountable Care Organizations in the Medicare Shared Savings Program. *JAMA*. 2025;333(21):1897-1905. doi:10.1001/jama.2025.3870.

© 2026 Khullar D et al. *JAMA Health Forum*.

Expenditures Included and Excluded in Calculations of Medicare Savings

	Included Spending Elements	Source and Comment	Excluded Spending Elements	Comment
Medicare Savings	Reduction in beneficiary health care spending relative to control	Per-beneficiary spending estimates multiplied by the total number of ACO-attributed beneficiaries<br>- Per-beneficiary spending estimates are derived from estimates in Bond et al (2025)[2]<br>- Number of ACO-attributed beneficiaries come from publicly reported data on MSSP performance[a]	Costs to CMS of administering MSSP	Unable to identify reliable MSSP administrative cost estimates for CMS
	Shared savings returned by CMS to ACOs (or excess spending on ACO patients kept by CMS)	Shared savings or shared costs come from publicly reported data on MSSP performance[a]		

[a] Centers for Medicare & Medicaid Services Data [Internet]. [cited 2025 Jan 28]. Performance Year Financial and Quality Results. Available from: https://data.cms.gov/medicare-shared-savings-program/performance-year-financial-and-quality-results

© 2026 Khullar D et al. *JAMA Health Forum*.
