# Supplementary material for: Budgetary Impact of the Medicare Shared Savings Program on Traditional Medicare
Source: JAMA Health Forum. 2026 Feb 20;7(2):e256915. doi: 10.1001/jamahealthforum.2025.6915 (PMC12924104; doi:10.1001/jamahealthforum.2025.6915)
Supplement: Supplement 2. — Data Sharing Statement [file jamahealthforum-e256915-s002.pdf]

Data Sharing Statement

Khullar. Budgetary Impact of the Medicare Shared Savings Program on Traditional Medicare. *JAMA Health Forum*. Published February 20, 2026. doi:10.1001/jamahealthforum.2025.6915

Data
Data available: No

Additional Information
Explanation for why data not available: Data are publicly available
